# A Dual Target-directed Agent against Interleukin-6 Receptor and Tumor Necrosis Factor α ameliorates experimental arthritis

**DOI:** 10.1038/srep20150

**Published:** 2016-02-04

**Authors:** Youngkyun Kim, Hyoju Yi, Hyerin Jung, Yeri Alice Rim, Narae Park, Juryun Kim, Seung Min Jung, Sung-Hwan Park, Young Woo Park, Ji Hyeon Ju

**Affiliations:** 1 CiSTEM laboratory, Convergent Research Consortium for Immunologic Disease, Seoul St. Mary’s Hospital, College of Medicine, The Catholic University of Korea, Seoul, 137-701, South Korea; 2 Division of Rheumatology, Department of Internal Medicine, Seoul St. Mary’s Hospital, College of Medicine, The Catholic University of Korea, Seoul, 137-701, South Korea; 3 Aging Research Center, Korea Research Institute of Bioscience and Biotechnology, Daejeon, 305-806, South Korea

## Abstract

A considerable proportion of patients with rheumatoid arthritis (RA) do not respond to monospecific agents. The purpose of our study was to generate a hybrid form of biologics, targeting tumor-necrosis factor alpha (TNFα) and interleukin-6 receptor (IL-6R), and determine its anti-arthritic properties *in vitro* and *in vivo*. A novel dual target-directed agent (DTA(A7/sTNFR2)) was generated by conjugating soluble TNF receptor 2 (sTNFR2) to the Fc region of A7, a new anti-IL-6R antibody obtained by screening the phage display human antibody library. DTA(A7/sTNFR2) inhibited the proliferation and migration of fibroblast-like synoviocytes from patients with RA (RA-FLS) more efficiently than single target-directed agents. DTA(A7/sTNFR2) also blocked osteoclastogenesis from bone marrow cells. The arthritis severity scores of the experimental arthritis mice with DTA(A7/sTNFR2) tended to be lower than those of mice with IgG, A7, or sTNFR2. Histological data suggested that DTA(A7/sTNFR2) is more efficient than single-target drugs in preventing joint destruction and bone loss. These results were confirmed *in vivo* using the minicircle system. Taken together, the results show that DTA(A7/sTNFR2) may be a promising therapeutic agent for the treatment of RA.

Rheumatoid arthritis (RA) is a common autoimmune disease characterized by chronic joint inflammation and progressive bone loss. Because the disease involves multiple genetic and environmental factors, treatment response varies and achieving a treatment that results in complete remission for all patients is difficult^1^. Many of the newer drugs for treating RA inhibit the molecular pathways responsible for the pathogenesis of the disease^2^. Biologic agents that target cytokines involved in the pathogenic signals contribute to revolutionary treatments for RA^3^.

Numerous proinflammatory cytokines are involved in the pathogenesis of RA, particularly tumor-necrosis factor alpha (TNFα) and interleukin-6 (IL-6)^4^. TNFα activates T-cells and induces T-cell infiltration and neoangiogenesis, and it leads to joint destruction by increasing proliferation of fibroblast-like synoviocytes (FLS) and formation of osteoclasts. IL-6 causes B-cells to proliferate and produce antibodies, and it also induces differentiation of T-cells into IL-17-secreting T-helper (Th17) cells, thereby suppressing regulatory T-cell differentiation. IL-6 has also been shown to stimulate angiogenesis and osteoclastogenesis^2^,^4^. Thus, TNFα and IL-6 likely contribute to many pathogenic signaling events that lead to RA.

Etanercept, a soluble TNF receptor 2 (TNFR2) conjugated with human IgG Fc region, and tocilizumab, a fully humanized anti-IL-6 receptor antibody, are the typical biological drugs approved by the FDA to treat RA. Etanercept blocks the binding of TNFα to TNF receptor 1 (TNFR1) and TNFR2 located on the cell surface^5^,^6^. Several clinical studies have shown that etanercept reduces disease activity more rapidly than methotrexate, the commonly used chemical drug for RA, in patients with RA^7^,^8^,^9^. Tocilizumab neutralizes IL-6 activity^10^,^11^ by binding to both the soluble IL-6 receptor (IL-6R) and the membrane bound IL-6R^12^, and its efficacy for the treatment of RA has been shown in many clinical trials^13^,^14^,^15^.

However, as with other drugs used to treat RA, etanercept and tocilizumab are ineffective in some patients^3^,^16^, which is thought to be due to the redundancy of the molecular pathway^4^. Like RA, cancer is a disease in which many cytokines are implicated^17^, and many new biologics that simultaneously target two molecules to avoid single-pathway resistance in cancer development have shown outstanding results in clinical trials^18^. For example, catumaxomab, which targets both epithelial cell adhesion molecule (EpCAM) and cluster of differentiation 3 (CD3) has been approved for the treatment of patients with EpCAM-positive cancer in Europe^19^.

Only four bispecific antibodies have been developed for RA treatment to date. Veri M.C. *et al.* reports the development of a dual-affinity re-targeting (DART) antibody that ameliorates arthritis in a collagen-induced arthritis (CIA) model by simultaneously binding to FcγRIIb and CD79B on the same B-cell, resulting in B-cell activation^20^. Three other bispecific antibodies reduced arthritis in CIA models: Kanakaraj P. *et al.* described a bispecific antibody that targets TNFα and angiopoetin 1^21^; Qi J. *et al.* described one against IL-1β and IL-17A^21^,^22^, and Liu M. *et al.* recently created one that targets TNFα and ED-B fibronectin^23^. These results suggest bispecific antibodies may be a promising drug for RA.

In this study, we developed a novel dual-target agent (DTA) composed of an anti-IL-6R antibody (A7) conjugated with sTNFR2 (DTA(A7/sTNFR2)). DTA(A7/sTNFR2) simultaneously binds to TNFα and IL-6R, potent cytokines involved in the pathogenesis of RA, with high affinity. DTA(A7/sTNFR2) inhibited osteoclastogenesis and impaired proliferation and migration in FLS *in vitro*, and ameliorated experimental arthritis in CIA models *in vivo*. Furthermore, we generated a minicircle vector encoding DTA(A7/sTNFR2) based on the biological characteristics of the agent and showed that this mode of administration was also effective at alleviating arthritis in CIA models. Taken together, we suggest that DTA(A7/sTNFR2) may be the basis for a novel drug to treat RA.

## Results

### Construction of DTA(A7/sTNFR2)

**Figure 1:**
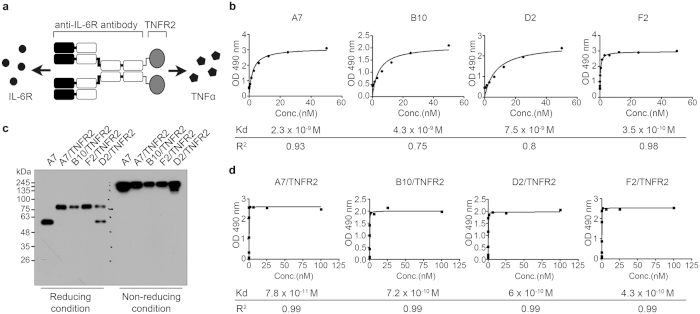
Generation of the dual target-directed antibody (DTA) against IL-6R and TNFα. (**a**) Diagram of DTA. DTA was constructed as an anti-IL-6R antibody conjugated with TNFR2 at the end of the Fc region. (**b**) Binding affinities of anti-IL-6R antibody candidates (A7, B10, D2, and F2) to IL-6R. The values of dissociation constant (Kd) and coefficient of determination (R^2^) are displayed under the graph. (**c**) Immunoblotting of DTA candidates (A7/TNFR2, B10/TNFR2, F2/TNFR2, and D2/TNFR2) with anti-Fc antibodies in reducing and non-reducing conditions. (**d**) Binding affinities of DTA candidates to TNFα. The values of Kd and R^2^ are displayed under the graph. All results are representative of at least three independent experiments.

Because signaling pathways of IL-6 and TNFα play key roles in RA pathogenesis, we designed a DTA against IL-6R and TNFα (Fig. 1a). First, we screened our phage display human antibody library to identify novel antibodies that bind to IL-6R with high affinity. After three rounds of panning, phages expressing anti-IL-6R antibodies were highly enriched (Supplementary Table S1), and monophage ELISA was performed using the enriched phage pool to select specific phage clones with anti-IL-6R antibodies (Supplementary Fig. S1). We identified 38 clones that carried one of the four different anti-IL-6R antibodies through sequencing (Supplementary Table S2 and Supplementary Fig. S2). The nucleotide sequences of these four candidate antibodies (A7, B10, F2, and D2) were cloned into mammalian expression vectors, and the antibodies were purified using these vectors (Supplementary Fig. S3). The dissociation constants of the candidates were less than 1 × 10^−8^ M (Fig. 1b).

To generate a DTA, the nucleotide sequences of sTNFR2 were attached to the 3′ end of the sequences encoding the constant region of the antibody heavy chain. The nucleotide fragments were introduced into the pNATAVH vector, which encodes heavy chain variable region of one of the four anti-IL-6R antibody candidates (Supplementary Fig. S4). The resulting vectors encoding four different DTAs (A7/TNFR2, B10/TNFR2, F2/TNFR2, and D2/TNFR2) were transfected into HEK293E cells, and antibodies were purified from the conditioned media of the cells. We used Western blotting with an anti-Fc antibody to confirm that all four DTAs were expressed in the HEK293E cells transfected with vectors encoding DTA (Fig. 1c). A7 was used as a positive control to show the heavy chain size in the intact IgG was about 50 kDa in the reducing condition and about 150 kDa in the non-reducing condition. The bands of the DTA candidates were detected higher than that of A7 in the reducing condition, indicating that their size is larger than that of original IgG due to addition of TNFR2. To distinguish the DTA with the highest affinity for TNFα, we tested the binding affinities of each DTA for TNFα (Fig. 1d). The dissociation constant of A7/TNFR2 was the lowest (7.8 × 10^−11^ M); therefore, we used this agent [DTA(A7/sTNFR2)] for further studies. Because the Kd of DTA(A7/sTNFR2) for IL-6R and TNFα were both low, DTA(A7/sTNFR2) was presumed to block IL-6 and TNFα signaling simultaneously.

### Inhibitory effects of DTA(A7/sTNFR2) on the production of inflammatory mediators induced by TNFα and the IL-6 signaling pathway

**Figure 2:**
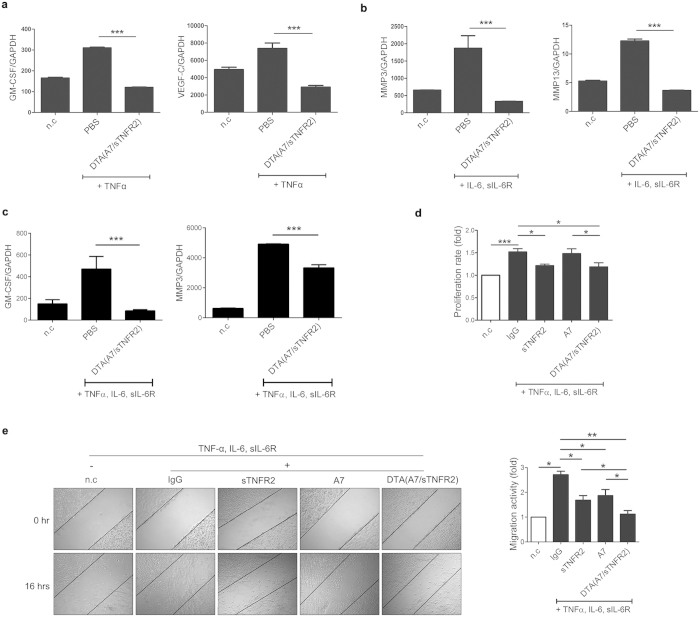
The inhibitory effects of DTA(A7/sTNFR2) on the production of inflammatory mediators and proliferation, and migration of RA-FLS. The inhibitory effects of DTA(A7/sTNFR2) on TNFα-induced GM-CSF and VEGF-C expression (**a**) or IL-6-induced MMP3 and MMP13 expression (**b**) in RA-FLS. RA-FLS were incubated with or without human TNFα (**a**) or IL-6 and sIL-6R (**b**) for 24 h. RA-FLS was also treated with 100 μg/mL DTA(A7/sTNFR2) or PBS (as a negative control) during the incubation. The mRNA expression of GM-CSF and VEGF-C or MMP3 and MMP13 was analyzed by realtime RT-PCR in triplicate. All values were normalized to that of GAPDH (Mean ± SEM). **(c)** The inhibitory effects of DTA(A7/sTNFR2) on GM-CSF and MMP3 expression induced by both of TNFα and IL-6 in RA-FLS. RA-FLS was incubated with or without TNFα, IL-6 and sIL-6R for 24h. RA-FLS was also treated with or without 100 μg/mL DTA(A7/sTNFR2) or PBS (as a negative control) during the incubation. The mRNA expression of GM-CSF and MMP3 was analyzed by realtime RT-PCR in triplicate. All values were normalized to GAPDH (Mean ± SEM). (**d**) Anti-proliferative effects of DTA(A7/TNFR2) on RA-FLS. RA-FLS were incubated with or without human TNFα, IL-6, and soluble IL-6R(sIL-6R) for 72 h. 100 μg/mL of sTNFR2, A7, DTA(A7/sTNFR2), or IgG was added during the incubation. Cell proliferation was assessed using CCK8 assay. Proliferation rates were normalized to the negative control (n.c.) value. Values are means ± SEM. (**e**, left) Representative results of the scratch assay to determine the anti-migration effects of DTA(A7/sTNFR2) on RA-FLS. After wounds were formed with sterile pipette tips, cells (with the exception of n.c.) were incubated with IgG, sTNFR2, A7, or DTA(A7/sTNFR2) in the presence of TNFα, IL-6, and IL-6R for 16 h. Black lines indicate the boundaries of the wounds. (**e**, right) Migration activities. Migration activities were calculated as follows: migration activity = 1—(wounded area at 16 h)/(wounded area at 0 h). The values were normalized to n.c. Mean ± SEM is presented. All results are representative of at least three independent experiments. *P*-values were obtained from Student’s *t*-test (*p < 0.05; **p < 0.01; ***p < 0.001).

To confirm that DTA(A7/sTNFR2) blocks TNFα and IL-6 signaling pathway, we investigated the inhibitory effects of DTA(A7/sTNFR2) on the production of inflammatory mediators induced by TNFα or IL-6. As TNFα increased the expression of vascular endothelial growth factor-C (VEGF-C) and granulocyte-macrophage colony-stimulating factor (GM-CSF) in RA-FLS^24^,^25^,^26^, we examined the effects of DTA(A7/sTNFR2) on mRNA expression of VEGF-C and GM-CSF using realtime RT-PCR in RA-FLS stimulated with TNFα. The results show that DTA(A7/sTNFR2) effectively blocked TNFα signaling (Fig. 2a). As IL-6 induces the expression of MMP3 and MMP13 in RA-FLS^27^, we performed realtime RT-PCR to investigate the inhibitory effects of DTA(A7/sTNFR2) on mRNA expression of these genes in RA-FLS incubated with IL-6 and sIL-6R. The data show that DTA(A7/sTNFR2) also blocks IL-6 signaling (Fig. 2b). We also confirmed that mRNA expression of GM-CSF and MMP-3 stimulated by both of TNFα and IL-6 signaling are blocked by DTA (Fig. 2c).

### Anti-proliferative and anti-migration effect of DTA(A7/sTNFR2) on fibroblast-like synoviocytes from patients with RA

The anti-proliferative effects of DTA(A7/sTNFR2) were analyzed in fibroblast-like synoviocytes from patients with RA (RA-FLS) *in vitro*. TNFα is a key factor for the proliferation of synoviocytes, and synoviocyte hyperplasia is a key characteristic of RA^4^. sTNFR2-Fc(sTNFR2) and DTA(A7/sTNFR2) inhibited the proliferation of RA-FLS whereas A7 did not (Fig. 2d).

Because IL-6 and TNFα signaling are involved in the migration of fibroblasts^28^, we performed the scratch assay to examine the anti-migration function of our novel agents *in vitro* (Fig. 2e). The migration activity of cells treated with sTNFR2, A7, or DTA(A7/sTNFR2) was lower than that of cells treated with IgG (Fig. 2e), and the migration activity of cells treated with DTA(A7/sTNFR2) tended to be the lowest among all the conditions, suggesting DTA(A7/sTNFR2) was more efficient at blocking cell migration than single target-directed agents.

### Anti-osteoclastogenic activity of DTA(A7/sTNFR2) on murine bone marrow cells

**Figure 3:**
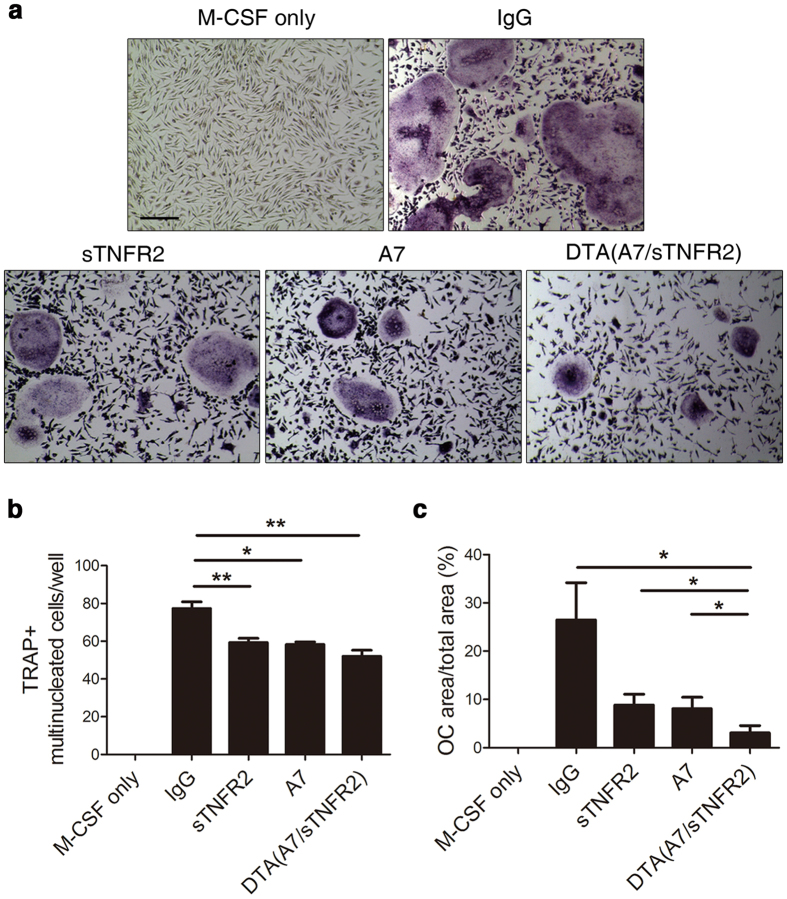
Anti-osteoclastogenic activity of DTA(A7/sTNFR2). (**a**) The representative data of TRAP staining. Osteoclast precursor cells were stimulated with M-CSF and RANKL and differentiated into osteoclasts. During the stimulation, cells were incubated with IgG, TNFR2, A7, or DTA (A7/sTNFR2). Cells were fixed and stained using a TRAP staining kit after 8 days of stimulation. Scale bar: 100 μm. (**b**) The number of osteoclasts. TRAP^+^ cells with eight or more nuclei were counted as osteoclasts. Mean ± SEM is presented. (**c**) The surface area of osteoclasts. The graph shows the percentage of osteoclast area (mean ± SEM). Cells treated with only M-CSF (M-CSF only) were used as a negative control for osteoclastogenesis. OC, osteoclast. All results are representative of at least three independent experiments. *P*-values were obtained from Student’s *t*-tests (*p < 0.05; **p < 0.01).

IL-6 and TNFα are involved in osteoclastogenesis, which is increased in RA and facilitates bone erosion^4^,^29^. We examined the anti-osteoclastogenic activity of DTA(A7/sTNFR2). The number of osteoclasts was lower in the cells treated with sTNFR2, A7, or DTA(A7/sTNFR2) than in the cells treated with IgG control (Fig. 3a,b). Furthermore, osteoclasts cultured with DTA(A7/sTNFR2) tended to be smaller than those in other groups (Fig. 3c).

### Amelioration of CIA by DTA(A7/sTNFR2)

**Figure 4:**
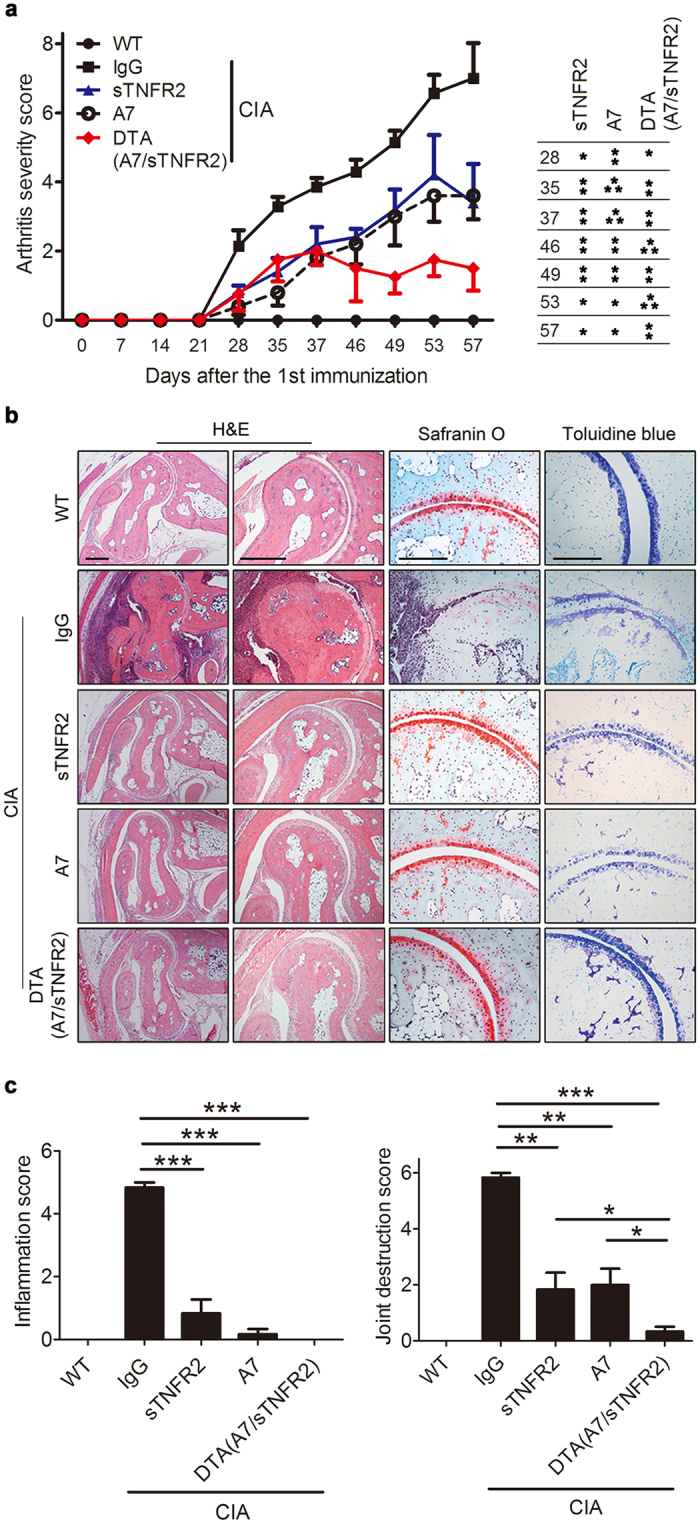
Amelioration of CIA by DTA(A7/sTNFR2). (**a**) Arthritis severity score of WT and CIA mice injected with IgG, sTNFR2, A7, or DTA(A7/sTNFR2). Results represent mean ± SEM. *P*-values (sTNFR2, A7, or DTA (A7/sTNFR2) vs IgG) are indicated as a table (**a**, right). (**b**) Representative histological images of the hind paws of WT mice and CIA mice injected with IgG, sTNFR2, A7, or DTA(A7/sTNFR2). Sections were stained with H&E, safranin O, or toluidine blue. Scale bars: 400 μm in the H&E images and 200 μm in the safranin O and toluidine blue images. (**c**) Inflammation and joint destruction scores measured based on the H&E and toluidine blue stains. Scores were evaluated by three independent observers. *P*-values were obtained from Student’s *t*-test (*p < 0.05; **p < 0.01; ***p < 0.001). WT, wild type; CIA, collagen-induced arthritis.

Arthritis continued to develop in the CIA group injected with IgG 21 days after the second IFA boost was administered (Fig. 4a), and the arthritis severity scores were lower in the CIA group injected with sTNFR2 or A7 than in those with IgG, which was consistent with previous studies^30^,^31^. The arthritis score tended to be lower in the DTA(A7/sTNFR2) than in the other groups.

To investigate the effects of DTA(A7/sTNFR2) on joints, hind limbs of mice were removed and stained with H&E, safranin O, and toluidine blue (Fig. 4b). According to the H&E data, the extent of pannus formation, synovial hyperplasia, and bone destruction was lower in the CIA groups treated with drugs than in the mice injected with IgG. The inflammation scores calculated based on H&E staining approached zero in the DTA(A7/sTNFR2)-treated group, suggesting the absence of arthritis symptoms in these mice (Fig. 4c). The safranin O and toluidine blue staining experiments showed arthritis-induced loss of cartilage was also lower in the DTA(A7/sTNFR2)-treated group (Fig. 4b). The H&E and toluidine blue staining showed the joint destruction scores were lower in the DTA(A7/sTNFR2) than in the other groups (Fig. 4c).

### Construction and expression of minicircle encoding DTA(A7/sTNFR2)

**Figure 5:**
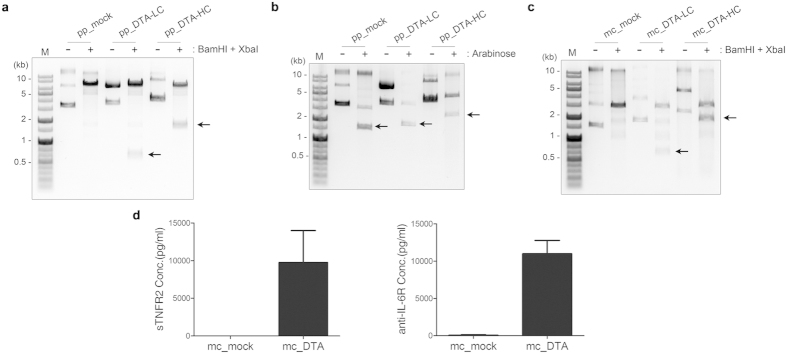
Generation of minicircles encoding DTA-LC and DTA-HC. (**a**) Gel electrophoresis data of parental plasmids digested with BamHI and XbaI. Arrows indicate the bands of the expected size for inserts. (**b**) Gel electrophoresis data of parental plasmids before and after arabinose induction. Arrows indicate the bands of the expected size for minicircles derived from parental plasmids. (**c**) Gel electrophoresis data of minicircles digested with BamHI and XbaI. Arrows indicate the bands of the expected size of the inserts. (**d**) The amount of DTA(A7/sTNFR2) in conditioned media of 293T cells transfected with mc_mock or mc_DTA (which includes mc_DTA-LC and mc_DTA-HC). The secretion of DTA(A7/sTNFR2) protein from the cells was assessed by ELISA for sTNFR2 and anti-IL-6R antibody (mean ± SEM). All results are representative of at least three independent experiments.

Although biologics like tocilizumab and etanercept are common drugs for the treatment of RA, they are expensive to prescribe, and the complex technology involved in synthesis and purification makes them time-consuming to prepare. Minicircles may be a more efficient, less expensive way to deliver biologics^32^. Because minicircles are vectors in which the bacterial backbone is removed, they are smaller than other conventional plasmids and their genes do not get silenced by bacterial DNA^33^,^34^,^35^. To examine whether DTA(A7/sTNFR2) is also effective in CIA models when it is introduced as a minicircle, we generated parental plasmids encoding the light and heavy chains of DTA(A7/sTNFR2) (referred to as pp_DTA-LC and pp_DTA-HC, respectively, Fig. 5a). Bands corresponding to the size of inserts were detected after enzyme restriction with BamHI and XbaI. We confirmed via electrophoresis that minicircles were generated after treatment with L-arabinose (Fig. 5b). Because the bacterial backbone of the parental plasmids was removed, the size of minicircles was smaller than that of parental plasmids (Fig. 5b; arrows). Inserts remained in the resulting minicircles (mc_DTA-LC and mc_DTA-HC, respectively), after enzyme restriction (Fig. 5c; arrows). Although the synthesis and folding of the antibody was complex, significant amounts of DTA(A7/sTNFR2) were detected in the conditioned media of HEK923T cells transfected with mc_DTA-LC and mc_DTA-HC (Fig. 5d).

### Amelioration of CIA by mc_DTA

**Figure 6:**
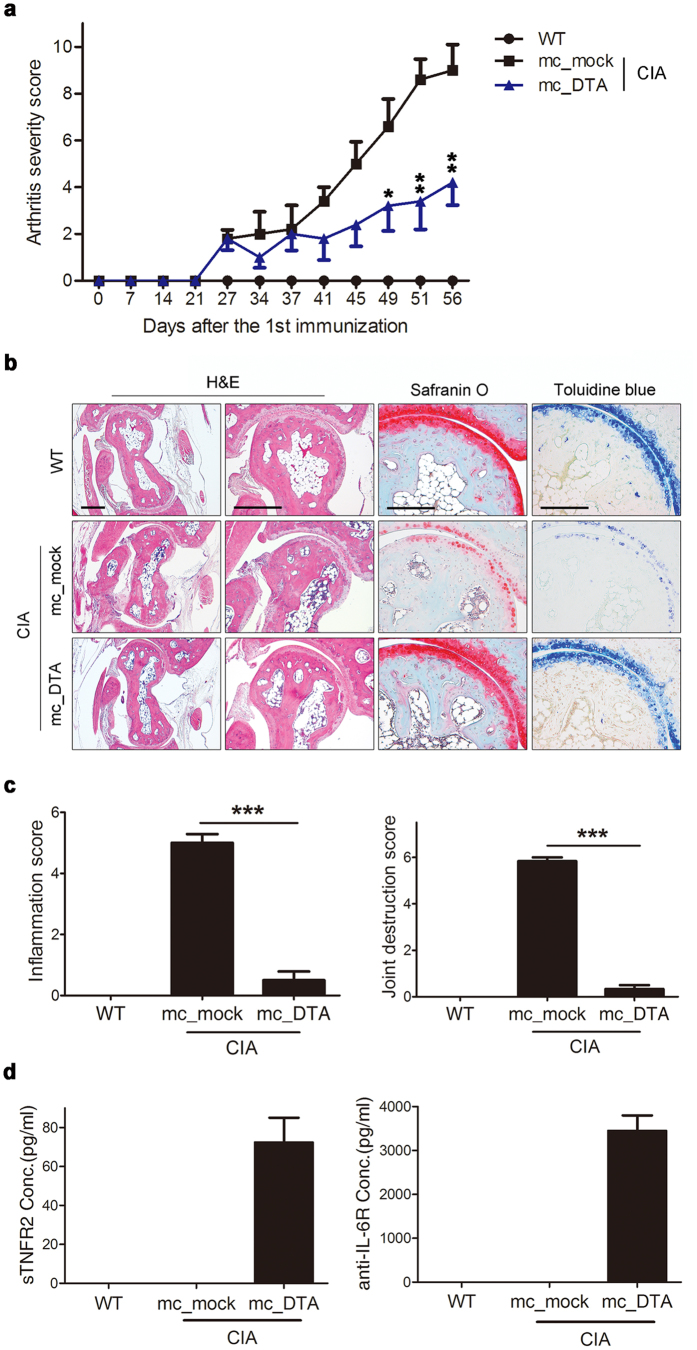
Amelioration of CIA with the injection of mc_DTA. (**a**) Arthritis severity score of WT and CIA mice injected with mc_mock or mc_DTA (mean ± SEM). *P*-values (mc_mock vs mc_DTA) are indicated. (**b**) Representative histological images of the hind paws of WT and CIA mice injected with mc_mock or mc_DTA. The sections were stained with H&E, safranin O, or toluidine blue. Scale bars: 400 μm in the H&E images and 200 μm in the safranin O and toluidine blue images. (**c**) Inflammation and joint destruction scores measured based on the H&E and toluidine blue stains. Scores were evaluated by three independent observers. (**d**) The amount of serum DTA(A7/sTNFR2) in the animals. The presence of the DTA molecule was assessed for sTNFR2 and anti-IL-6R antibody using ELISA (mean ± SEM). *P*-values were obtained from Student’s *t*-test (*p < 0.05; **p < 0.01; ***p < 0.001). All results are representative of at least three independent experiments. WT, wild type; CIA, collagen-induced arthritis.

To identify the effects of mc_DTA, we injected CIA mice intravenously with mc_mock or mc_DTA and monitored arthritis severity scores (Fig. 6a). Similar to intraperitonieal injection of DTA(A7/sTNFR2) (Fig. 4a), the minicircle injection of mc_DTA ameliorated arthritis in the CIA mice. H&E, safranin O, and toluidine blue staining of hind limbs showed that the mc_DTA efficiently blocked joint destruction, including pannus formation and loss of cartilage (Fig. 6b). The inflammation and joint destruction scores of the mice injected with mc_DTA were much lower than those injected with mc_mock (Fig. 6c). To assess whether the amelioration of CIA was associated with protein expression of DTA(A7/sTNFR2) derived from mc_DTA, serum DTA(A7/sTNFR2) were calculated using ELISA for sTNFR2 and anti-IL-6R antibody (Fig. 6d). The mc_DTA-injected mice expressed significantly greater amounts of sTNFR2 and anti-IL-6R antibody, suggesting DTA(A7/sTNFR2) was synthesized by the host.

## Discussion

Dual-targeted antibodies have introduced a novel mechanism to treat complex diseases^36^,^37^. Bispecific antibodies, which bind two different epitopes of a molecule, block the target with higher affinity than monospecific antibodies. Moreover, bispecific antibodies, which target two molecules that utilize different signaling pathways, overcome drug resistance caused by signaling redundancy. Although treatment with two monospecific antibodies would have the same effects as a bispecific antibody, the two monospecific antibodies would be more than double the cost^38^. Bispecific antibodies can have special functions in addition to masking the target molecules, making them attractive for drug development. For example, blinatumomab, a bispecific T-cell engager (BiTE) that targets CD19 and CD3, connects T-effector cells to transformed B-cells^39^. Catumaxomab selectively lyses tumor cells by acting as a bridge for CD3-expressing T-cells, EpCAM-expressing tumor cells, and Fc receptor-expressing accessory cells, such as dendritic cells, macrophages, and NK cells^19^.

In this study, we developed a novel DTA that targets IL-6R and TNFα simultaneously. Because these cytokines are critical players in the pathogenesis of RA^1^, we hypothesized that blocking both IL-6 and TNFα signaling may be a good strategy to treat RA. Therefore, we obtained four new anti-IL-6R antibodies by screening a phage display human antibody library (Fig. 1b) and conjugated these antibodies with sTNFR2 to produce DTAs. DTA(A7/sTNFR2) had the highest affinity for TNFα (Fig. 1d) and effectively inhibited proliferation and migration of FLS and osteoclastogenesis of osteoclast precursor cells (Figs 2 and 3). When DTA(A7/sTNFR2) was introduced into CIA mice, pathological symptoms, such as pannus formation, joint destruction, and loss of cartilage, were clearly weaker than in the CIA mice given IgG control antibodies (Fig. 4). Moreover, the efficacy of DTA(A7/sTNFR2) tended to be better than the single target-directed agents sTNFR2 and A7. We injected the same amount of drugs (100 μg) in all groups. Because the molecular size of DTA(A7/sTNFR2) is much larger than the size of sTNFR2 or A7, the efficacy of DTA(A7/sTNFR2) was likely better than an equimolar concentration of sTNFR2 or A7, respectively.

DTA(A7/sTNFR2) likely had a higher efficacy than monospecific drugs due to its dual targeting characteristics. TNFα is abundant in serum and synovial fluid of patients with RA^40^, and many clinical studies have shown that blocking TNFα signaling effectively treats RA^8^. However, a significant number of patients do not respond to these treatments^16^, which may be explained in part by the diverse basal level of serum TNFα in patients with RA^41^,^42^. IL-6, another cytokine abundant in patients with RA, has similar characteristics as TNFα, and can also induce RA^4^. Development of arthritis was synergistically blocked in TNFRI and IL-6 double knockout mice, inferring a complementary relationship between TNFα and IL-6 43. Furthermore, clinical studies show that tocilizumab attenuates RA in patients who do not respond to TNF blockers^44^,^45^. These data suggest the simultaneous binding to both cytokines by DTA(A7/sTNFR2) may help treat RA in patients who have high levels of both TNFα and/or IL-6.

We also showed that using the minicircle system to administer DTA(A7/sTNFR2) to CIA mice was effective. Antibodies are successful drugs because of their specificity and are easy to design with the advent of gene engineering. However, they are expensive to synthesize, purify, and quality-test *in vitro* and *in vivo*, which may hinder the development of new biologics in small scale laboratories. Recently, we suggested that minicircles may be a cheap and efficient way to develop and qualify novel biologics^32^. To confirm the effects of DTA(A7/sTNFR2) using this system, we generated minicircles encoding DTA(A7/sTNFR2) and injected them into mice through hydrodynamic tail vein injections. As expected, DTA was synthesized *in vivo* and arthritis was attenuated in the mice with DTA(A7/sTNFR2), further indicating the effectiveness of DTA(A7/sTNFR2) in CIA mice and the usefulness of the minicircle system for testing the efficacy of new biologics.

We confirmed that DTA(A7/sTNFR2) was synthesized from mc_DTA *in vitro* (Fig. 5d) and *in vivo* (Fig. 6d) using ELISA for sTNFR2 and anti-IL-6R antibody. As DTA(A7/sTNFR2) consists of equal amounts of epitopes for both ELISA, similar amounts of sTNFR2 and anti-IL-6R antibody are expected to be measured as in Fig. 5d. However, serum concentration of sTNFR2 in mc_DTA-treated CIA mice was much lower than that of anti-IL-6R antibody (Fig. 6d). We assumed that it was due to DTA bound to TNFα. Considering the difference of concentration was detected only in serum, we think DTA bound to transmembrane IL-6R is not able to be in serum because it sticks to cell surfaces (Supplementary Fig. S5; cases of II and IV). On the other hand, DTA bound to TNFα exists in serum because TNFα is a soluble protein (Supplementary Fig. S5; case of III). If sTNFR2 ELISA can’t detect DTA bound to TNFα efficiently, the concentration of DTA analyzed by sTNFR2 ELISA will be different from that analyzed by anti-IL-6R ELISA. To test whether this assumption is correct, various amounts of TNFα were incubated with 20 ng/ml of DTA for 2 hrs at room temperature followed by the sTNFR2 ELISA. As a result, the concentration of DTA detected by sTNFR2 ELISA was lower at the higher concentration of TNFα incubated with DTA (Supplementary Fig. S5).

Although we have shown that DTA(A7/sTNFR2) may be a promising drug for RA, many aspects still need to be studied for the clinical application, including how long they are stable *in vivo* and how effectively they can localize to inflamed joints. This information would clarify whether DTA has the adverse effects of anti-TNFα blockers and tocilizumab, such as serial infection and tumorigenesis, and clarify the possible mechanism of DTA(A7/sTNFR2), such as translocation of transmembrane TNFα-expressing macrophages to joints with elevated soluble IL-6R or the existence of reverse signaling induced by DTA-bound transmembrane TNFα. Elucidating the mechanism of DTA(A7/sTNFR2) will help future modifications of the drug to improve its efficiency.

To our knowledge, we are the first to develop a novel DTA that simultaneously targets TNFα and IL-6, the most important cytokines involved in the pathogenesis of RA. DTA(A7/sTNFR2) effectively inhibited proliferation and migration of RA-FLS and osteoclast formation in osteoclast precursors. DTA(A7/sTNFR2) also appeared to ameliorate experimental arthritis more efficiently than single target-directed agents in CIA mice, which was confirmed by the minicircle system. Thus, DTA(A7/sTNFR2) may be a promising drug for the treatment of RA.

## Materials and Methods

### Mice

Female DBA1/J mice (5–6 weeks of age) were purchased from OrientBio (Seongnam, Korea) and housed in specific pathogen-free conditions. All procedures involving animals were in accordance with the Laboratory Animals Welfare Act, the Guide for the Care and Use of Laboratory Animals, and the Guidelines and Policies for Rodent Experimentation provided by the Institutional Animal Care and Use Committee (IACUC) of the School of Medicine at the Catholic University of Korea. This study protocol was approved by the institutional review board of The Catholic University of Korea (CUMC-2011-0062-03).

### Cells

HEK293E and HEK293T cells were maintained in Dulbecco’s Modified Eagle Medium (DMEM; Gibco) supplemented with 7.5% fetal bovine serum (FBS; Gibco), 100 U/mL penicillin, and 100 μg/mL streptomycin. FLS from RA patients (RA-FLS) were obtained from rheumatoid synovectomy tissues and were maintained in DMEM supplemented with 10% FBS, 100 U/mL penicillin, and 100 μg/mL streptomycin. This study protocol about human FLS was approved by the institutional review board of The Catholic University of Korea (KC12TISI0861).

### Western blot

HEK293E cells transfected with pNATABL and pNATABH encoding A7 or DTA candidates were harvested and lysed with lysis buffer (1% NP40 [w/v], 1 mM phenylmethylsulphonyl fluoride, and 10 μM leupeptin in PBS) for 40 min at 4 °C. Cell lysates were centrifuged at 12,000 × g for 20 min at 4 °C and supernatants were collected. Sample buffer was added to the samples and boiled for 10 min. Sample buffer for reducing condition was 50 mM Tris-HCl (pH 6.8), 5% 2-mercaptoethanol, 2% SDS (w/v), 0.1% bromophenol blue (w/v), and 10% glycerol (w/v). Sample buffer without 2-mercaptoethanol was used for the non-reducing condition. The protein samples were separated by SDS-PAGE and transferred to nitrocellulose membranes. Membranes were blocked with PBS containing 5% skim milk for 1 h and incubated with horseradish peroxidase-conjugated anti-human IgG Fc antibody (Thermo Scientific) at a dilution of 1:5,000 for 1 h at room temperature. After washing with PBS containing 0.1% Tween 20 (PBS-T), the membrane was treated with the enhanced chemiluminescence reagent WESTSAVE UP (Ab frontier) and the signals were measured using a Fusion SL imaging system (Viber Lourmat)

### Binding affinity test

ELISA was used to investigate binding affinities of candidates for anti-IL-6R antibody and DTA. A total of 100 ng human IL-6R-Fc or TNFα was placed in wells on a 96-well plate overnight at 4 °C. After blocking with 2% skim milk in PBS for 2 h at room temperature, serially diluted antibody candidates (from 50 nM to 0.02441 nM) were added to the wells and incubated for 2 h. The wells were washed three times with PBS-T, and HRP-conjugated anti-Fab antibody (Abcam) was added to the wells at a dilution of 1:4,000 in 2% skim milk (100 μL per well). After incubation for 1 h, the wells were washed with PBS-T and treated with *o*-phenylenediamine dihydrochloride for 7 min to detect HRP. After HRP detection, 50 μL of 1 N H_2_SO_4_ was added. Optical density (OD) values were measured at 490 nm.

### Realtime RT-PCR

RA-FLS were incubated with or without human TNFα (20 ng/mL; R&D systems) for 24 h and were also incubated with PBS or 100 μg/mL DTA. Another set of RA-FLS was incubated with or without human IL-6 (100 ng/mL; R&D systems) and human soluble IL-6R (100 ng/mL; PeproTech) for 24 h. PBS or 100 μg/mL DTA were added to the cells during the incubation. After incubation, RNA was prepared using Trizol (Life Technologies) according to the manufacturer’s instructions. The expression levels of human VEGF-C, GM-CSF, MMP3, and MMP13 were evaluated by realtime RT-PCR using LightCycler 480 SYBR green I master kit (Roche) and LightCycler 480 (Roche). The sequences of primers used are shown below; for VEGF-C (NCBI reference sequence: NM_005429.4), 5′-AGGCCAACCTCAACTCAAGG-3′and 5′-TCGCGACTCCAAACTCCTTC-3′; for GM-CSF (NM_000758.3), 5′-GAGACACTGCTGCTGAGATGA-3′and 5′-AGGGCAGTGCTGCTTGTAG-3′; for MMP3 (NM_002422.3), 5′-AGCCAACTGTGATCCTGCTT-3′and 5′-CCACGCCTGAAGGAAGAGAT-3′; for MMP13 (NM_002427.3), 5′-GAGCTGGACTCATTGTCGGG-3′and 5′-CTGCATTTCTCGGAGCCTCT-3′.

### Cell proliferation assay

RA-FLS were incubated with or without human IL-6 (100 ng/mL; R&D systems), human soluble IL-6R (100 ng/mL; PeproTech), and human TNFα (20 ng/mL; R&D systems) for 72 h. During the incubation, RA-FLS were treated with 100 μg/mL of sTNFR2, A7, DTA(A7/sTNFR2), or IgG. RA-FLS were also cultured without cytokines and antibodies as a negative control. Cell proliferation was assessed using the cell counting kit-8 (CCK-8; Dojindo Molecular Technologies) according to the manufacturer’s instructions.

### Scratch assay

RA-FLS were seeded onto 6-well plates (Corning-Coastar). When the RA-FLS reached 100% confluency, the monolayer was scratched with a sterile 200-μL tip and the cells were cultured with 1.5 mL fresh DMEM supplemented with 100 U/mL penicillin and 100 μg/mL streptomycin. Human IL-6 (200 ng/mL), human soluble IL-6R (200 ng/mL), and human TNFα (20 ng/mL) were added to the media of all but the negative control cells. During the incubation with cytokines, RA-FLS were also treated with 100 μg/mL sTNFR2, A7, DTA(A7/sTNFR2), or IgG. Phase-contrast microscopy images were acquired at 0 and 16 h after the wounds were created. The migration activities were calculated as follows: migration activity = 1- (wounded area at 16 h)/(wounded area at 0 h).

### Osteoclastogenesis

Bone marrow cells were isolated from the femurs and tibias of 7-week-old DBA1/J mice and exposed to ACK buffer (0.15 mM NH_4_Cl, 1 mM KCO_3_, and 0.1 mM EDTA, pH 7.4) for 10 min to deplete red blood cells. The cells were washed with α-minimum essential medium (α-MEM; Gibco) supplemented with 10% FBS containing 100 U/mL penicillin, and 100 μg/mL streptomycin. After the cells were cultured in α-MEM overnight, the nonadherent cells were cultured for 2 days in the presence of 10 ng/mL murine macrophage colony-stimulating factor (M-CSF; Peprotech). The medium was changed to fresh α-MEM containing 10 ng/mL murine M-CSF and 30 ng/mL murine receptor activator of nuclear factor kappa-B ligand (RANKL; Peprotech), along with IgG (10 μg/mL), sTNFR2 (10 μg/mL), A7 (10 μg/mL), or DTA(A7/sTNFR2) (10 μg/mL) 2 and 5 days after the first treatment with M-CSF. On day 8, the cells were fixed and analyzed with tartrate-resistant acid phosphatase (TRAP) staining using a commercial kit (Sigma) as described by the manufacturer except without hematoxylin counterstaining. TRAP^+^ multinucleated cells with eight or more nuclei were counted as osteoclasts.

### Induction and assessment of CIA

Bovine type II collagen (CII; Chondrex) was dissolved in 0.1 M acetic acid at a concentration of 2 mg/mL and incubated at 4 °C overnight. CII was emulsified 1:1 with complete Freund’s adjuvant (CFA) containing 2 mg/mL heat-killed *Mycobacterium tuberculosis* (Chondrex). Female DBA1/J mice (5–6 weeks of age; OrientBio) were injected intradermally at day 0 with 100 μL CII/CFA emulsion. The same concentration of CII emulsified with incomplete Freund’s adjuvant (IFA; Chondrex) was injected into mice as a booster immunization 21 days after the first immunization. Five mice were used in the wild-type group and seven were used in each of the CIA groups (IgG, sTNFR2, A7, DTA, mc_mock, or mc_DTA-injected group, respectively). The drugs (100 μg/mouse; approximately 5 mg per kg of body weight) were given to the animals intraperitoneally three times per week for 3 weeks starting 24 days after the first immunization. The severity of arthritis was monitored and scored as described previously^46^.

### Histological evaluation of arthritis

Mice were sacrificed 57 days after the first immunization. The hind limbs were fixed in 10% formalin and decalcified in 10% (w/v) EDTA, followed by embedding in paraffin. The 4-μm-thick sections were stained with hematoxylin and eosin (H&E), safranin O, or toluidine blue. The inflammation score and joint destruction score were measured according to the procedure of Huckel *et al.*^47^. A higher inflammation score represented more synovial hyperplasia and infiltration of leukocytes. A higher joint destruction score represented more pannus formation and erosion of cartilage. At least three sections for each group were analyzed and three independent observers graded all scores blindly.

### Production of minicircles

The minicircles mc_mock, mc_DTA-LC, and mc_DTA-HC were produced as described by Kay *et al.*^48^. Briefly, *E. coli* ZYCY10P3S2T cells were transformed with the parental plasmids and grown overnight at 37 °C in Terrific Broth containing 50 μg/mL kanamycin. The cultures were combined with LB broth containing 0.02% l-(+)-arabinose and incubated at 30 °C for 5 h. Minicircle DNA was isolated using the Nucelobond Xtra Midi kit.

### *In vivo* gene delivery

Minicircles were delivered to mice 18 days after CIA induction using the hydrodynamic tail vein injection method^49^. Briefly, 8 μg mc_DTA-LC and 12 μg mc_DTA-HC were injected together into the mc_DTA mice to produce DTA(A7/sTNFR2) antibodies *in vivo*, while 20 μg mc_mock was injected into the mc_mock group of mice. Minicircles were injected in a normal saline solution at about 10% of the mouse body weight. To increase the hydrodynamic pressure, 27-gauge needles were used and injections were performed within 5–7 seconds. Five mice were used in each group.

### Detection of DTA derived from minicircles

HEK293T cells were transfected with minicircle vectors using the Lipofectamine 2000 reagent according to the manufacturer’s instructions. Both mc_DTA-LC and mc_DTA-HC were transfected into HEK293T cells to generate DTA(A7/sTNFR2)-expressing cells. The mc_mock was transfected into HEK293 cells as a negative control. To determine the amount of DTA(A7/sTNFR2) expressed by the transfected cells, the culture media was analyzed 24 h post-transfection using ELISA. DTA(A7/sTNFR2) was quantified using human sTNF-R (80 kDa) platinum ELISA (eBioscience), and the anti-IL-6R antibody was detected using sandwich ELISA, as previously described^32^. Ten days after the injection of minicircles, 100 μL venous blood was collected from the orbital sinus of anesthetized mice, and the serum DTA(A7/sTNFR2) was analyzed as described above.

### Statistical analysis

Statistical analysis was performed using Student’s *t*-tests. *P* < 0.05 was considered significant.

## Additional Information

**How to cite this article**: Kim, Y. *et al.* A Dual Target-directed Agent against Interleukin-6 Receptor and Tumor Necrosis Factor α ameliorates experimental arthritis. *Sci. Rep.* **6**, 20150; doi: 10.1038/srep20150 (2016).

## Supplementary Material

Supplementary Information
