# Supplementary material for: A Dual Target-directed Agent against Interleukin-6 Receptor and Tumor Necrosis Factor α ameliorates experimental arthritis
Source: Sci Rep. 2016 Feb 4;6:20150. doi: 10.1038/srep20150 (PMC4740770; doi:10.1038/srep20150)
Supplement: Supplementary Information [file srep20150-s1.pdf]

Supplementary information

A Dual Target-directed Agent against Interleukin-6 Receptor and Tumor Necrosis Factor α effectively ameliorates experimental arthritis

Youngkyun Kim[†1], Hyoju Yi[†1], Hyerin Jung[1], Yeri Alice Rim[1], Narae Park[1], Juryun Kim[1], Seung Min Jung[1,2], Sung-Hwan Park[2], Young Woo Park[3] and Ji Hyeon Ju[1,2].

[†]These authors contributed equally to this work.

[1]*CiSTEM laboratory, Convergent Research Consortium for Immunologic Disease, Seoul St. Mary's Hospital, College of Medicine, The Catholic University of Korea, Seoul, 137-701, South Korea.*

[2]*Division of Rheumatology, Department of Internal Medicine, Seoul St. Mary's Hospital, College of Medicine, The Catholic University of Korea, Seoul, 137-701, South Korea.*

[3]*Aging Research Center, Korea Research Institute of Bioscience and Biotechnology, Daejeon 305-806, South Korea*

***Corresponding author:**

Ji Hyeon Ju, M.D., Ph.D.

CiSTEM laboratory, Convergent Research Consortium for Immunologic Disease, Division of Rheumatology, Department of Internal Medicine, Seoul St. Mary's Hospital, College of Medicine, The Catholic University of Korea, #505, Banpo-Dong, Seocho-Gu, Seoul, South Korea.

Telephone: 82-2-2258-6013; Fax: 82-2-3476-2274;

E-mail address: juji@catholic.ac.kr

Supplementary tables

Table S1. Rates of phages binding IL-6R after each round of panning.

	**1st round**	**2nd round**	**3rd round**
**Phage input (cfu)**	8.5×10^{13}	2.78×10^{13}	2.3×10^{13}
**Phage output (cfu)**	3.1×10^{6}	1.69×10^{7}	8.2×10^{8}
**Output/input ratio**	3.6×10^{-8}	6.1×10^{-7}	3.6×10^{-5}

cfu: colony forming unit.

Table S2. The sequences of complementarity determining regions (CDR) of heavy chain (upper table) and light chain (lower table) of anti-IL-6R antibodies.

	**CDR H1**	**CDR H2**	**CDR H3**
**A7**	DYAMH	GVSWNSGTIAYVDSVKG	DFTYFYESSGYYAFDL
**B10**	DYAMF	GINWNGNGIGYGDSVRG	PSLYGGNSEFDL
**D2**	NYAIN	RIIPMLGTSDYAEKFQG	GPRYYGTDSYYLEK
**F2**	NYYMH	IINPSGGNTGYAQKFQG	GLPWGENGLDV

	**CDR L1**	**CDR L2**	**CDR L3**
**A7**	TGTNSNIGAGYDVH	GNTNRPS	QSFDSSLT
**B10**	TGPTIGAGYDVH	GNLNRPS	HTYDSSLS
**D2**	TGPTIGAGYDVH	GNLNRPS	HTYDSSLS
**F2**	TGSSSNIGAGYDVH	GDSDRPS	QSYDSSLS

Supplementary figures

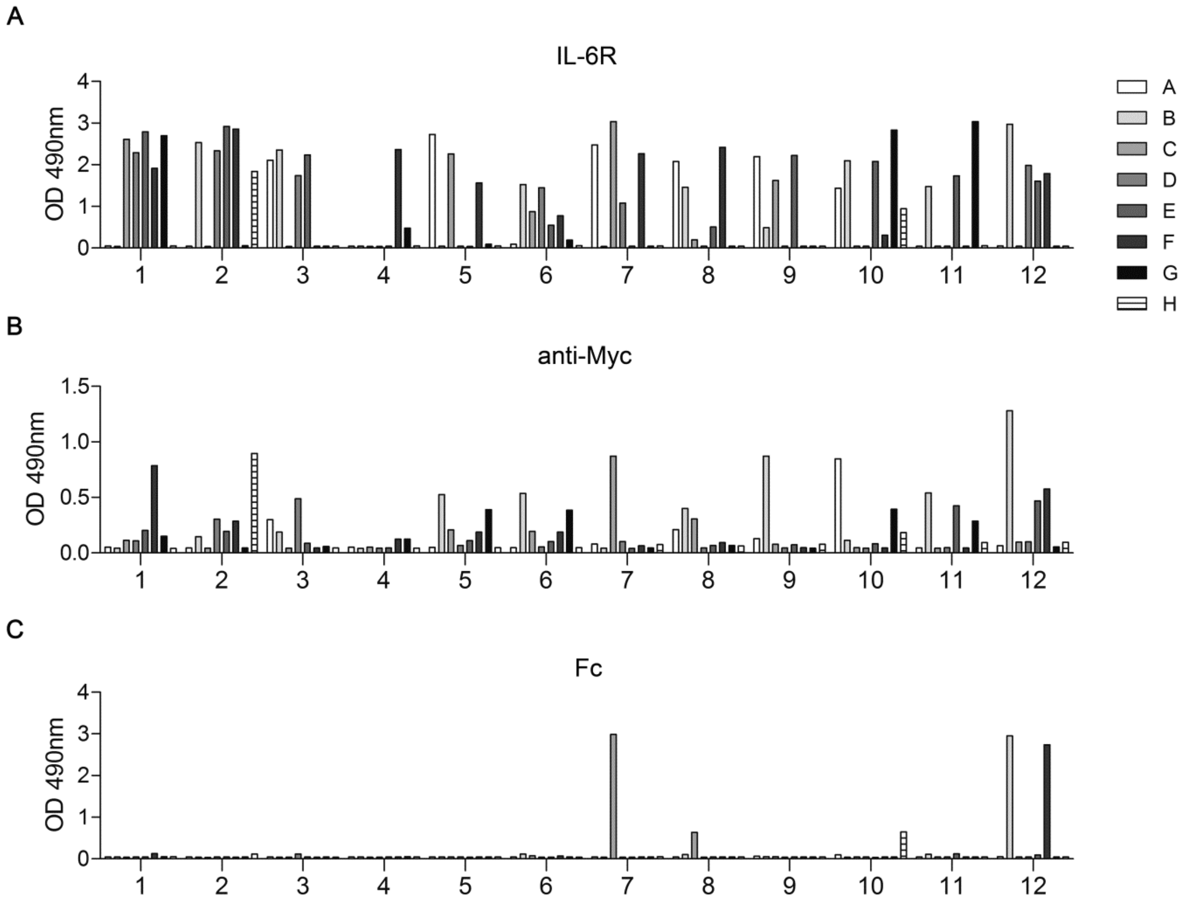

Fig. S1. Monophage ELISA results. The OD values of each well of the 96-well plates which are coated with IL-6R (A), anti-Myc (B), or Fc (C) are indicated. The OD values of anti-Myc show that all of the 96 phages are amplified properly. There were 41 clones of which the OD value for the IL-6R-coated plate was more than 1.4. Among them, three clones of which the OD value for the Fc-coated plate was more than 1.4 were excluded for further studies. ELISA with a Fc-coated plate was conducted as a negative control because Fc region was conjugated the IL-6R protein which was used for panning.

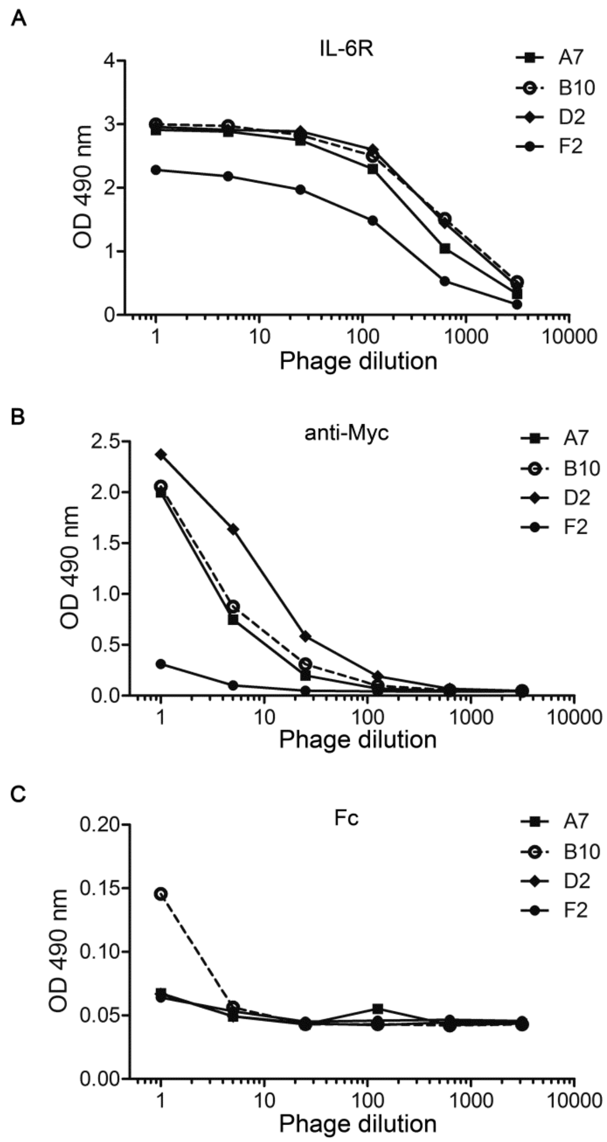

Fig. S2. Monophage dilution ELISA results. Four phages from the A7, B10, D2, and F2 well of 96-well plates were serially diluted (from 1:1 to 1: 3,125) and ELISA experiments were conducted with the IL-6R (A), anti-Myc (B), or Fc (C)-coated plates. The OD values were measured at 490nm. The results show that these phages bind to the IL-6R in a dose-dependent manner.

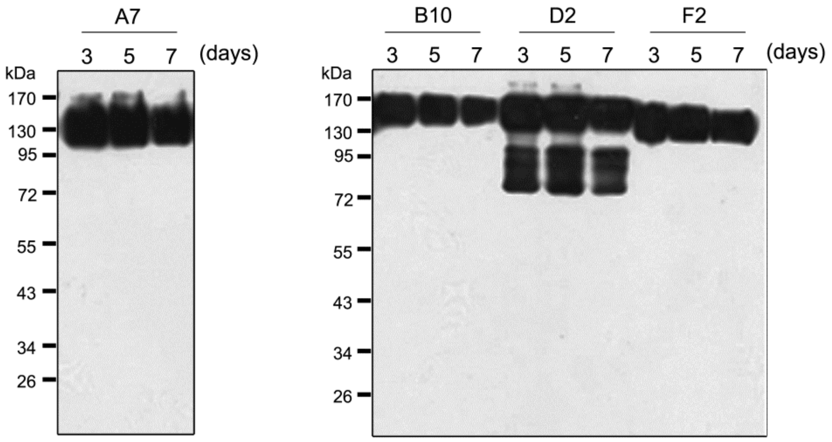

Fig. S3. Expression of anti-IL-6R antibodies from the conditioned media of 293E cells transfected with pNATABH and pNATABL which encodes the sequences of anti-IL-6R antibody candidates. Western blotting was performed with horseradish peroxidase-conjugated anti-human IgG Fc antibody at a dilution of 1: 4,000. In non-reducing condition, bands for intact IgG (~150kDa) were detected.

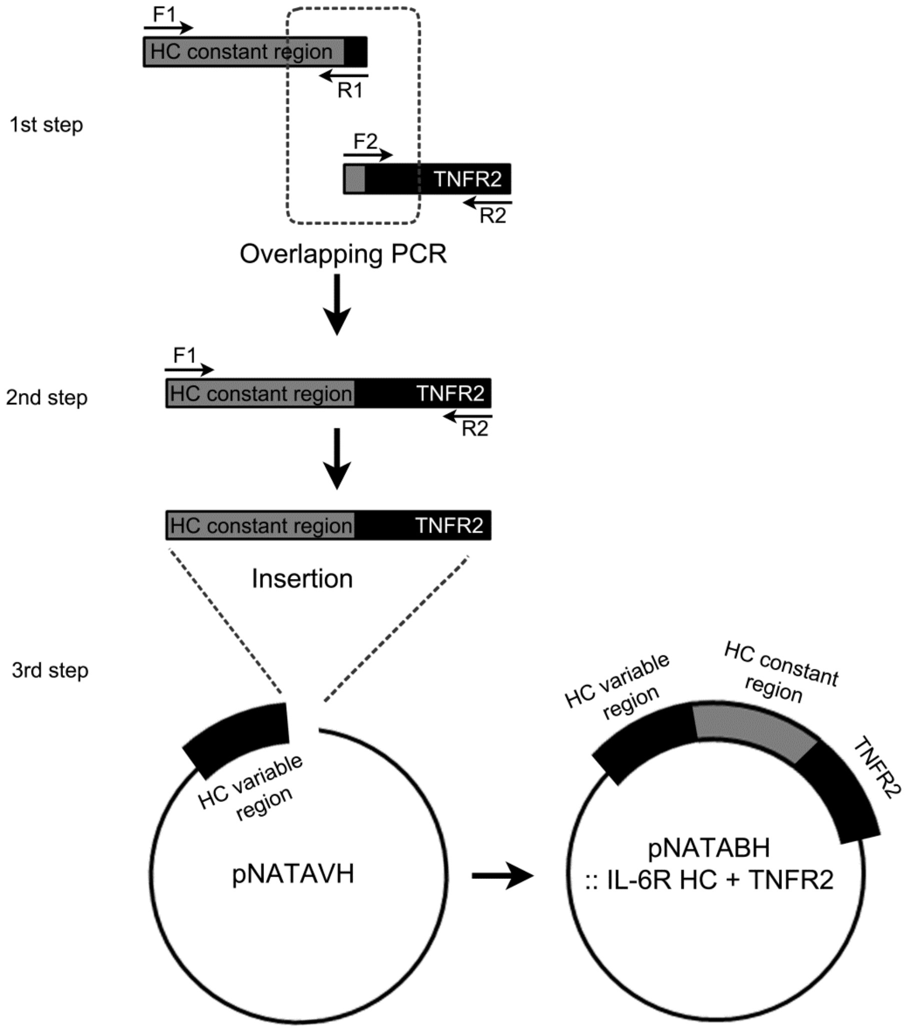

Fig. S4. Diagram of the construction of pNATABH encoding DTA heavy chain composed of heavy chain of anti-IL-6R antibody and TNFR2.

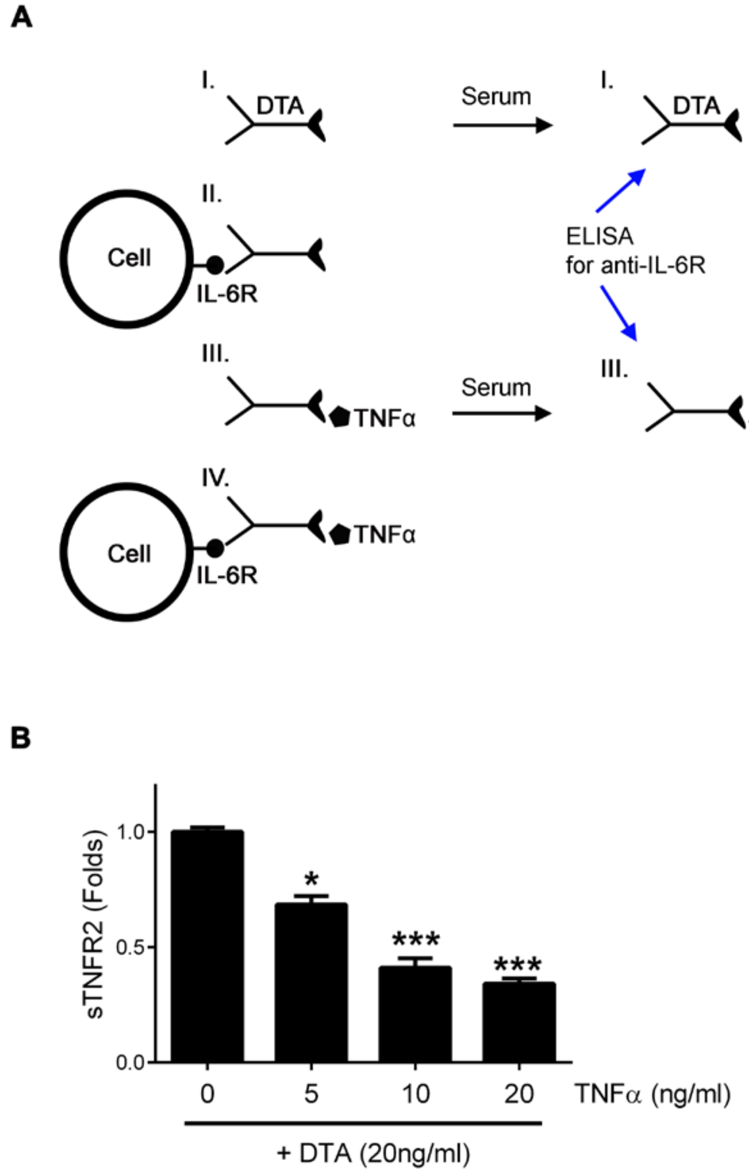

Fig. S5. Scheme and an ELISA result explaining why the differences in serum ELISA for sTNFR2 and anti-IL-6R antibody exist. **(A)** As DTA bound to transmembrane IL-6R is attached to cell surface (case of II and IV), DTA bound to TNFα only (case of III) or none (case of I) may exist more than DTA bound to IL-6R in serum. If ELISA for sTNFR2 cannot detect DTA bound to TNFα efficiently, the concentration of DTA analyzed by ELISA for sTNFR2 will be lower than that by ELISA for anti-IL-6R antibody. **(B)** The concentration of DTA analyzed by ELISA for sTNFR2 after various concentrations of TNFα was incubated with 20 ng/ml of DTA for 2 hrs at room temperature (mean ± SEM). *P*-values were obtained

from Student's *t*-test (*, $p<0.05$; ***, $p< 0.001$). ELISA experiments were performed independently three times.

Supplementary methods

Phage rescue and panning. A scFv library which was developed by KRIBB was used for anti-IL-6R antibody screening. Phage rescue from *Escherichia coli* XL1-Blue (Stratagene) containing phagemids was performed by helper phage VCS-M13 (Stratagene). Phage panning was carried out according to standard protocols (1, 2). Briefly, the library was incubated in the immunotube coated with IL-6R-Fc at room temperature for 2 hrs. After serial washing with PBS-T(0.1 % Tween 20 in PBS), the bound phages were eluted with 100 mM triethylamine and amplified by infecting and growing XL1-Blue in 2X YT media (16 g Bacto-tryptone, 10 g Bacto-yeast extract, 5 g NaCl in 1 L distilled water) supplemented with 4% glucose and 10 mM $MgCl_2$. Three rounds of panning were performed.

Monoclonal phage Enzyme-linked immunosorbent assay (ELISA). After three rounds of panning, 96 clones were picked and grown in the 96-well plates with 2X YT media containing 4 % glucose and 10 mM $MgCl_2$ overnight at 37℃. VCS-M13 helper phages were added to each well, and incubated for 30 min at 37℃. After spinning at 3,200 x g for 20 min, supernatant were discarded and pellets were incubated with 2X YT media supplemented with 10 mM MgCl2, 1 mM isopropyl-β-d-thiogalactosid, and 50 μg/ml Kanamycin overnight at 30℃. After centrifugation at 3,200 x g for 20 min at 4℃, the supernatants were used for monoclonal phage ELISA. Briefly, 100ng of IL-6R-Fc, anti-Myc antibody (Abcam), or Fc was coated into each well of 96-well plates. IL-6R-Fc and Fc were generated and purified from 293E cells transfected with mammalian expression vector pYK604 encoding IL-6R-Fc and Fc, respectively. After blocking with 2 % skim milk in PBS for 2 hrs, the phage

supernatant was introduced into the wells. After washing with PBS, HRP-conjugated anti-M13 antibody was added at dilution 1:2,000 in 2 % skim milk (100 μL per well). After 50 min later, the wells were washed with PBS, and the *o*-phenylenediamine dihydrochloride (OPD) was added to detect HRP. Stop solution (1N H_2SO_4) was added and OD values were measured at 490 nm.

Construction of pNATABH encoding DTA. To generate mammalian expression vector encoding DTA, nucleotide sequence of sTNFR2 was attached to 3' end of human IgG heavy chain constant region through overlapping PCR following standard protocols (3). The resulting DNA fragment was inserted into the site right after heavy chain variable region in pNATAVH through cloning using restriction enzymes. The resulting vector was named as pNATABH. As there were four candidates for anti-IL-6R antibody, there were four different pNATABH vectors encoding four different DTA; A7/sTNFR2, B10/sTNFR2, D2/sTNFR2, and F2/sTNFR2.

Synthesis and purification of antibodies and sTNFR2. To synthesis and purify DTA, pNATABH was co-transfected into the HEK293E cells with pNATABL, which is a vector carrying human IgG light chain of the candidate for anti-IL-6R antibody, by Lipofectamine 2000 (Invitrogen). Cells were cultured to become fully confluent and the conditioned media were collected. DTA were purified from these conditioned media using protein A sepharose beads (Sigma-aldrich) following the manufacturer's instructions. Anti-IL-6R antibodies including A7 antibody were produced as described above using pNATABH without sTNFR2 sequences and pNATABL. sTNFR2 was also generated with same methods except that the nucleotides of sTNFR2 were delivered in pYK602 vector.

Construction of pp_DTA. Plasmids pNATABH_DTA-HC and pNATABL_DTA-LC encoding DTA(A7/sTNFR2) heavy and light chains, respectively, were constructed from AR&T. The sequences of DTA-LC and DTA-HC were amplified by PCR from those plasmids using the following oligonucleotide primers: 5′–TCTAGAGCCACCATGCAGCTCGTGCTGACTCAGCCGCCCTCAG–3′ and 5′–GGATCCTTCCTCAGAGATCAGCTTCTGCTCTCA–3′ for DTA LC, and 5′–TCTAGAGCCACCATGGGATGGAGCTATATCATCCTCTTTTTGGTG –3′ and 5′–GGATCCTTCCTCAGAGATCAGCTTCTGCTCTCA–3′ for DTA HC. All primers included restriction sites for XbaI or BamHI, to allow subsequent digestion for cloning into the parental plasmid pMC.CMV-MCS-EF1-GFP-SV40PolyA, which was purchased from System Biosciences. The parental plasmids, including pp_mock, pp_DTA-LC, and pp_DTA-HC, were isolated using the Nucleobond xtra midi kit (Macherey-Nagel).

Evaluation of sensitivity of sTNFR2 ELISA. TNFα at various concentrations (0, 5, 10, or 20 ng/ml) was incubated with 20 ng/ml DTA for 2 hrs at room temperature. The concentration of DTA was assessed using human sTNF-R (80 kDa) platinum ELISA (eBioscience) according to the manufacturer’s instruction.

1. Lee CM, Iorno N, Sierro F, Christ D. Selection of human antibody fragments by phage display. Nat Protoc 2007;2:3001-8.

2. Ridgway JB, Ng E, Kern JA, Lee J, Brush J, Goddard A, et al. Identification of a human anti-CD55 single-chain Fv by subtractive panning of a phage library using tumor and nontumor cell lines. Cancer Res 1999;59:2718-23.

3. Bryksin AV, Matsumura I. Overlap extension PCR cloning: a simple and reliable way to create recombinant plasmids. Biotechniques 2010;48:463-5.
